# Ageing with HIV

**DOI:** 10.3390/healthcare6010017

**Published:** 2018-02-14

**Authors:** Padraig McGettrick, Elena Alvarez Barco, Patrick W. G. Mallon

**Affiliations:** 1 HIV Molecular Research Group, UCD School of Medicine, University College Dublin, Dublin, Ireland; elena.alvarezbarco@ucd.ie (E.A.B.); paddy.mallon@ucd.ie (P.W.G.M.); 2 Mater Misericordae University Hospital, Eccles street, Dublin 7, Ireland

**Keywords:** HIV, ageing, non-AIDS illness, frailty, cardiovascular disease, malignancy, cognitive impairment, inflammation

## Abstract

The population of people living with HIV (PLWH) is growing older with an estimated 4 million over the age of 50 years, a figure which has doubled since the introduction of effective antiretroviral therapy (ART) and which is increasing globally. Despite effective ART, PLWH still experience excess morbidity and mortality compared to the general population with increased prevalence of age-related, non-AIDS illnesses (NAI) such as cardiovascular disease, malignancies, cognitive impairment and reduced bone mineral density, which impact disability and everyday functioning. This review will discuss the challenges presented by comorbidities in ageing PLWH and discuss the aetiology and management of age-related illnesses in this vulnerable population.

## 1. Introduction

Since the introduction of combination antiretroviral therapy (ART), HIV has transitioned from a progressive and often terminal illness to a chronic manageable disease, with people living with HIV (PLWH) achieving life expectancy approaching that of the general population [1,2]. This improvement in life expectancy and longevity has seen an increase in the number of PLWH living into older age, with an estimated 4 million people currently living with HIV aged over 50, a figure which has doubled since the introduction of ART and is continuing to increase globally [3,4]. Despite effective virological control PLWH still experience excess morbidity and mortality compared to the general population [5]. Age-related illnesses such as cardiovascular disease, malignancies, osteoporosis and cognitive impairment are all more prevalent in an ageing population of PLWH with some occurring at an earlier age [5]. With increasing prevalence of co-morbidities, come new and additional challenges, with a switch in emphasis to incorporate management of age-related, non-AIDS illnesses (NAI). An understanding of these NAI and the often-complex interplay with HIV infection is necessary in order to optimise health outcomes in an aging cohort of PLWH and reduce the potential disability associated with them. 

This review will focus on six comorbidities commonly seen in an ageing population, namely cardiovascular disease, malignancy, cognitive impairment, osteopenia/osteoporosis, frailty and disability but which are, as will be discussed, more prevalent in PLWH and will likely increase with an ageing demographic. It will summarise major areas of ongoing research into the aetiology of NAI with a particular focus on inflammation and its role in the development of comorbidities and will discuss the implications but also the limitations of research to date in HIV and ageing. 

## 2. Methods

A literature search of peer-reviewed publications up to August 2017, through Medline, was conducted following search keywords: ‘HIV’ or ‘human immunodeficiency virus’ AND ‘ageing,’ ‘Cardiovascular disease,’ ‘malignancy’ OR ‘non-AIDS malignancy,’ ‘osteoporosis’ OR ‘osteopenia’ OR ‘reduced BMD,’ ‘neurocognitive impairment’ OR ‘cognitive impairment’ OR ‘HIV Associated neurocognitive disorder,’ ‘Frailty’ OR ‘Disability,’ ‘Inflammation’ OR ‘immune activation.’ Eligibility criteria included English language articles, published in peer reviewed journals with preference given to articles citing most recent, or historically most clinically significant data. Case reports, articles pertaining to a paediatric or young adult population and the treatment of AIDS related conditions were excluded. Other sources were also searched including the Conference of retroviruses and Opportunistic Infections (CROI) online database of conference proceedings for years 2015 to 2017 inclusive, Clinicaltrials.gov online database, UNAIDS and governmental publications (accessed online July 2017). 

All relevant articles were independently reviewed by two authors (McGettrick and Alvarez). Studies were selected for inclusion based on eligibility criteria above and relevance to topics discussed as defined by three broad sub-headings: epidemiology, pathogenesis and treatment/management. Differences were resolved by consensus with preference given to articles citing most recent data and based on study design, for example, when overlapping data existed between cohort studies, the study with the largest population or longest follow up was included or, in the instance of updated cohort data, the paper citing most recent data was included. When significant conflicting data within topics existed, the authors attempted to include and reflect this in the manuscript. Selected articles were then reviewed by third author (Mallon) to assess for inclusion validity and relevance to topics discussed. Final agreement on articles included was by consensus of three authors as of August 2017.

## 3. Results

**Figure 1:**
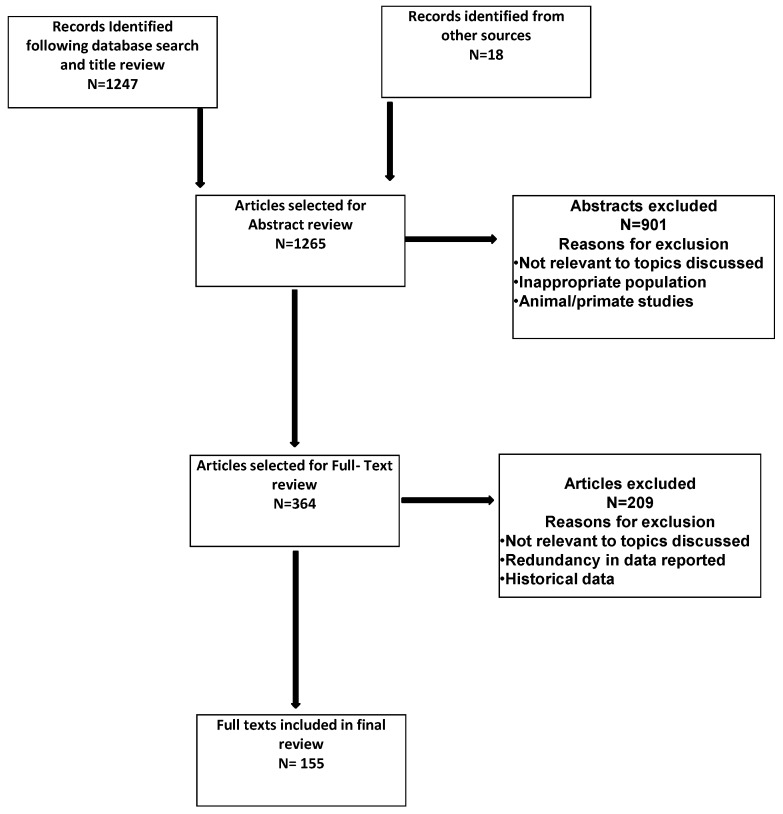
Flow chart of literature search.

1247 articles were selected for abstract review following initial search results, title review and exclusion of duplicates. Following abstract review, a further 364 were selected for full text review with 147 of these included in the final review based on analysis criteria outlined above (Figure 1). 8 references from other sources are also included in the final review. 

## 4. Summary of Evidence

### 4.1. Cardiovascular Disease

PLWH are at increased risk of developing cardiovascular disease (CVD) compared to the general population and this risk appears to persist despite effective virological suppression [6,7]. While the risk of death associated with CVD is reducing in the general population, recent data on mortality from the United States suggests it may be increasing in PLWH [8], although this has not been observed in other cohort studies [9]. However, CVD is estimated to account for 11–30% of mortality in PLWH [10,11] and while there is a relatively high prevalence of traditional CVD risk factors in populations of PLWH, this does not adequately account for the observed higher prevalence [12,13,14]. 

Chronic inflammation has been linked to the development of CVD in PLWH with those not on ART with detectable virus more likely to develop CVD than those who are on effective, fully suppressive ART [7,15]. Higher circulating markers of inflammation, such as hsCRP and IL6, are associated with increased prevalence of, and worse outcome with, CVD in PLWH [16,17]. It has also been shown that vascular inflammation occurs earlier in PLWH despite effective ART [18] with PLWH more likely to develop high risk atherosclerotic plaques compared to HIV negative controls matched for age and traditional risk factors [19]. Antiretroviral therapy reduces but does not normalise the circulating inflammatory markers suggesting a persistent chronic inflammatory state despite ART, the causes of which will be discussed below. 

The role of ART in the development of CVD in PLWH remains the subject of debate. While there is little doubt that ART, by reducing HIV viremia and inflammation, reduces the overall risk of CVD [7], certain antiretroviral medications, such as the nucleoside reverse transcriptase inhibitor (NRTI) abacavir and some protease inhibitors (PI), have been associated with an increased risk of CVD in some studies [20] but is not a consistent observation, with uncertainty remaining as to magnitude of additional risk attributable to ART exposure. 

Current use, or use in the last six months of abacavir has been associated with an increased risk of myocardial infarction (MI) [21]. This association, although repeatedly observed in a number of observational cohort studies, has not consistently been observed in randomised, clinical trials and at least two meta-analyses [22,23]. Despite this, guidelines recommend considering alternatives to abacavir in PLWH with established CVD or presence of multiple risk factors [24,25].

It is known that certain antiretroviral regimes can alter traditional risk factors, including induction of dyslipidaemia and in this way, may contribute to CVD risk. The non-nucleoside reverse transcriptase inhibitor efavirenz has been shown to increase total cholesterol, LDL and HDL levels when compared to a regimen containing an integrase inhibitor [26] while the nucleotide reverse transcriptase inhibitor tenofovir disoproxil fumarate (TDF) appears to reduce atherogenic lipids through a mechanism that remains unclear [27]. Despite these effects on lipids, however, exposure to neither efavirenz or TDF modified CVD risk when adjusted for alterations in lipid levels [28,29]. Cumulative exposure to NRTI has also been linked to development of insulin resistance [30]. First generation protease inhibitors such as indinavir and saquinavir, although rarely used currently, are also known to affect serum lipid concentrations, with cumulative exposure associated with additional CVD risk [31]. More recent protease inhibitors, such as atazanavir and darunavir, used frequently in contemporary clinical practice, are associated with less dyslipidaemia but there is emerging data that darunavir exposure is also associated with an increased risk of MI [32]. As these data emerge, clinicians treating PLWH are better able to limit the impact of ART on CVD risk by avoiding use of specific ART drugs in PLWH at higher risk of CVD.

Like in the general population, risk calculators for CVD are used to help identify PLWH who would benefit from primary preventative therapies such as HMG Co-Enzyme A reductase inhibitors or ‘Statins.’ However, CVD risk calculators have not been validated in cohorts of PLWH and are thought to underestimate actual risk of CVD in this cohort [33]. In addition, use of statins, although shown to reduce both serum lipid levels and development of high risk coronary plaque features, in both HIV negative and positive populations [34], is often limited in PLWH by significant interactions with concurrent ART. Pitavastin, a recent addition to the statin family, does not utilise the cytochrome p450 system for metabolism and therefore reduces the number of potential drug-drug interactions with ART. It has been shown to be efficacious in PLWH with dyslipidaemia and is currently being studied as an intervention to prevent the development of CVD in PLWH who are deemed low to moderate risk based on traditional CVD risk factors [35,36]. Other therapeutics targeting inflammation in PLWH are also being developed such as Canakinumab, a novel anti-IL1b monoclonal antibody, which has shown some promising early results [37]. 

Considerable gaps remain in our knowledge of the relative contribution of ART, inflammation and traditional risk factors to the development of CVD in PLWH. A recent study in the United States that modelled projected incidence of CVD in PLWH found that HIV carried a similar risk for the development of CVD as diabetes mellitus and suggested HIV infection be considered as a major CVD risk factor [38]. As the cohort of PLWH ages, CVD is likely to remain a significant cause of morbidity and mortality and HIV-specific interventions to reduce this risk are urgently needed to optimise health outcomes.

### 4.2. Malignancy

Since the introduction of ART, the prevalence of AIDS defining cancers (ADC) such as Kaposi Sarcoma, primary CNS lymphoma, non-Hodgkin’s lymphoma and invasive cervical carcinoma, have reduced with improved HIV virological control and immune recovery, although still occurring at increased frequency compared to the general population [39]. However, PLWH on ART with good restoration of their CD4^+^ T cell count remain at increased risk of all cancers compared to the general population, including non-AIDS defining cancers (NADC) such as lung, liver, head and neck and anal carcinoma [39,40]. It is estimated that approximately 30% of mortality in PLWH is attributable to malignancies and, at cancer diagnosis, PLWH are more likely to have a more advanced and aggressive disease [41,42,43,44]. With the introduction of ART there has been a marked reduction in ADCs, however NADCs such as Hodgkin’s lymphoma appear to be increasing [45]. The most important risk factors for the development of NADC include increasing age, length of time since diagnosis of HIV, smoking and co-infection with certain oncogenic viruses such as Epstein Barr virus, Human Papillomavirus, Human Herpes virus 8 and Hepatitis B and C virus [46,47,48]. The role of ART in the development of NADC is controversial with some studies reporting an association between exposure to certain classes of ART and the development of NADC such as anal carcinoma and Hodgkin’s lymphoma [49,50]. However, this has not been consistently observed and given the known beneficial effects of ART on inflammation and immune recovery, further studies are needed to clarify if this may be due to the life prolonging effects of ART rather than to any directly oncogenic effects [51].

Residual immune dysfunction despite viral suppression on ART has also been implicated in the development of NADC in PLWH. Markers of chronic inflammation and coagulation are associated with increased cancer risk in PLWH [50,52,53,54]. Recent epidemiological studies suggest that PLWH with a lower current CD4^+^ T cell count appear to be more at risk of developing NADC than those with higher counts [55,56]. Meta-analyses comparing cancer incidence in PLWH and immunosuppressed transplant patients reported similar incidence and pattern of malignancies in these two groups, highlighting immunodeficiency as an important factor in the development of these malignancies [39]. A lower nadir CD4^+^ T cell count has also been shown to have an inverse relationship with the development of NADC, again suggesting that residual immune dysfunction despite restoration of peripheral CD4^+^ T cell counts may be important in the pathogenesis of NADC [50,57]. Although in a large proportion of PLWH, ART is effective at restoring peripheral CD4^+^ T cell count, this inadequately reflects the often irreversible and persistent disruption and damage present in lymphoid tissue despite effective viral suppression [58]. Whether current recommendations, advising early commencement of ART regardless of CD4^+^ T cell counts with expectant relative preservation of immune function, will positively impact on rates of NADC remains to be seen. 

Regardless of the underlying aetiology, clinicians must be watchful for signs and symptoms of potential malignant processes in aging PLWH. Screening for certain malignancies such as cervical carcinoma and hepatocellular carcinoma (HCC) in hepatitis B and C co-infected patients should be performed routinely in HIV clinics. However, with an aging population we are faced with ensuring adequate screening protocols to include screening for a range of additional NADC in order to reduce the considerable morbidity and mortality resulting from malignancy in PLWH, a fact reflected in current European HIV management guidelines [24]. 

### 4.3. Osteopenia/Osteoporosis

The prevalence of both low bone mineral density (BMD) and osteoporosis increases with age and PLWH are at increased risk of developing low BMD compared to age-matched, HIV negative controls, often also being diagnosed with low BMD at a younger age [59,60,61]. This increased risk of osteoporosis translates into clinically-relevant fracture risk, with increased rates of fractures associated with low BMD observed in PLWH. These fractures can have significant impact on daily function and can lead to increased disability [62,63,64,65,66]. Reductions in BMD observed in PLWH are related to HIV infection itself [59,61,67], the relative high prevalence of traditional risk factors for low BMD such as smoking and low body weight [59] as well as exposure to ART [60,68,69], which plays a significant and complex role in the development of low BMD and osteoporosis in PLWH.

In the first year following initiation of ART there is a significant decrease in BMD detectable at both the spine and hip [70]. This reduction in BMD is accompanied by increased markers of bone resorption and formation, suggesting increased bone turnover, the mechanism of which remains unclear [71]. However, following the first year after ART initiation there appears to be a plateauing of BMD to a new baseline, albeit one lower than pre-ART initiation, on a background of persistently higher bone turnover. Most studies have shown a relative stability of BMD thereafter suggesting a long-term benefit of suppressive ART despite the early reduction in BMD at initiation [72,73,74,75,76] but with smaller changes in BMD with some ART switches [77,78,79,80]. Importantly, with ART initiation, recommencement of ART as second line therapy after virological failure with first line treatment also results in loss of BMD suggesting that the losses observed are, at least in part, arising through the period of control of HIV viremia with ART [81].

One medication which has been consistently linked with reductions in BMD at initiation is the TDF, a pro-drug of active tenofovir [82,83,84]. However, tenofovir alafenamide, an alternative formulation prodrug that leads to much less systemic exposure to active tenofovir, does not appear to be associated with the same reductions in BMD suggesting that systemic tenofovir exposure likely contributes to greater BMD loss at ART initiation [85]. The use of anti-resorptive therapies such as bisphosphonates in the setting of HIV and osteoporosis do increase BMD, however larger studies are needed to demonstrate whether these changes result in reductions in clinical fracture rates [86,87,88]. Novel strategies to reduce the initial reductions in BMD following initiation of ART, such as bisphosphonate use at initiation, have shown some promising results, with prospective, placebo controlled studies ongoing, the outcome of which may inform clinical practice in the future and may aid in reducing the morbidity and disability associated with osteoporosis in this cohort [89,90]. 

### 4.4. Cognitive Impairment

Cognitive impairment is another important issue in an ageing population of PLWH and one which can have a significant impact on disability. HIV was first noted to be associated with cognitive dysfunction, not related to opportunistic infections, early in the AIDS epidemic [91,92]. HIV Associated Neurocognitive Disorders (HAND) is the term used to describe a spectrum of neurological dysfunctions observed in PLWH, ranging from asymptomatic cognitive impairment (ACI) on neuro-psychological testing to AIDS Dementia Complex (ADC), a severe cognitive dysfunction affecting memory and concentration as well as displaying motor and behavioural changes. Rates of ADC, strongly associated with advanced HIV infection with low CD4^+^ T cell counts and high HIV RNA levels in cerebrospinal fluid (CSF), have significantly reduced since the introduction of ART in the mid-nineties [93]. Although ART, by suppressing HIV replication, has dramatically reduced the risk of developing the severe presentation of ADC it does not appear to have significantly reduced the prevalence of milder forms of HAND [93]. As the population of PLWH ages, there is a concern that the prevalence of HAND may increase, with older age a major risk factor for the presence of cognitive disorders including HAND in PLWH and evidence of a more rapid progression in PLWH despite effective treatment [94,95,96,97]. The prevalence of cognitive impairment in the ART era remains high, with the majority of cases classified as asymptomatic cognitive impairment (ACI) [98]. Whether these asymptomatic cases progress to functional impairment has been questioned [99,100], however recent longitudinal studies would suggest ACI, at the very least, represents a risk factor for developing symptomatic impairment although the progression appears somewhat variable [101,102].

The precise pathogenesis of HAND remains unclear but is likely multifactorial. HIV enters the CNS early in the course of infection and although cannot infect neurons directly, infects microglia and macrophages, which can stimulate a local inflammatory response that indirectly results in the production of neurotoxins and neuronal loss. It has been shown that despite effective ART, markers of inflammation within the CNS remain elevated in PLWH [103,104]. Why this is the case remains unclear. The CNS may act as a reservoir for persistent, low-level HIV replication in the setting of ART, with discordance in the virological suppression between serum and CSF samples thought to be due to variable penetrance of specific ART regimes across the blood brain barrier. However, ART has also been implicated in the development of features of HAND. Efavirenz, a NNRTI with well documented neurological side effects, in a multivariate analysis, was found to be associated with increased risk of cognitive impairment [105]. In Vitro research examining the neurotoxicity of ART demonstrated a direct toxic effect on rat neurons of commonly used antiretrovirals at equivalent doses to that used for the treatment of HIV, although the degree of toxicity was generally modest [106].

Other potential causes of persistent neuroinflammation include gut microbial translocation. Damage to the gut-associated lymphoid tissue (GALT) occurs early in HIV infection leading to increased microbial translocation across the gut wall and into the systemic circulation, resulting in further activation of the innate immune system. Migration of these activated monocytes across the blood brain barrier may contribute to neuroinflammation and subsequent damage [107,108,109]. Both lower nadir CD4^+^ T cell count and occurrence of an AIDS illness have also been identified as risk factors for the development of HAND in both the pre- and post-ART eras, suggesting that despite successful viral suppression and at least partial restoration of immune function with ART, there may be a persistent immune dysfunction contributing to the development of cognitive impairment in some PLWH [93,110,111]. 

Whatever its cause, HAND remains a serious concern for an aging cohort of PLWH. Some have advocated screening of patients for cognitive impairment, identifying asymptomatic impairment as a risk factor for the development of symptomatic disease [101]. However, there is a lack of consensus and more research is needed into both its pathogenesis and also into potential strategies to reduce the prevalence of cognitive dysfunction. With advancing age and increasing longevity, this will undoubtedly remain and likely increase, as a significant cause of morbidity and disability in PLWH. 

### 4.5. Frailty and Disability

With the emergence of non-AIDS defining illnesses as a major cause of morbidity and mortality in ageing PLWH on successful ART, immune and virological markers traditionally used in the monitoring of HIV such as CD4^+^ T cell count and HIV RNA may not be useful in predicting those at risk of these age-related conditions [112]. In addition, age, while an important factor, cannot be solely relied upon, as people of similar ages often differ dramatically in terms of presence of co-morbidities and general ill health. The concept of ‘frailty’ describes a vulnerability to adverse outcomes in the face of stressors and in the HIV negative population this ‘phenotype’ has been associated with increased disability arising from falls, hospitalisations and reduced mobility as well as morbidity and overall mortality [113,114]. Cohort studies in PLWH have shown frailty to be more prevalent and occurring at a younger age in PLWH compared to HIV negative populations [115,116,117]. In PLWH, frailty has also been shown to be predictive of all-cause mortality, morbidity and progression to AIDS [117,118,119]. In the ART era, frailty has been associated with lower current CD4^+^ T cell counts and longer time since HIV diagnosis as well as lower nadir CD4^+^ T cell count and a lower CD4^+^ CD8^+^ T cell ratio despite effective therapy [117,120,121,122]. There is some evidence that frailty scores may be more useful in predicting all-cause mortality in PLWH on treatment than traditional treatment markers such as CD4^+^ T cell count and HIV RNA. 

However, the definition of and scores used to measure, frailty vary considerably in these studies making comparisons difficult. Most frailty studies use the definition for frailty proposed by Fried et al. focusing on five domains, weakness (as measured by grip strength), slowness (as measured by walking test), weight loss (as measured by direct measurement), low energy/exhaustion (self-report) and low physical activity (as calculated by weeks energy expenditure). However, some large cohort studies, such as Multicentre AIDS Cohort Study (MACS) use a modified version of this, while others vary measurement of some of the domains by self-report and direct measurement. The heterogeneity of frailty definitions and measurements used in these studies make it difficult to draw firm conclusions with regards the clinical utility of frailty scores in clinical practice and further, large studies are needed. 

Whereas the concept of frailty describes a vulnerability to adverse outcomes when faced with stressors, disability refers to difficulty completing daily tasks and activities. Already there is evidence that PLWH are at increased risk of disability compared to the HIV negative population [123]. In fact, the reported rates of disability in cohort studies of PLWH are remarkably high, ranging from 18–39% in those aged over 50 [124,125]. Strong predictors of disability, such as cognitive impairment and increased risk of falls, as already discussed, occur at increased frequency in PLWH [126,127,128] meaning that in an ageing population of PLWH with increasing prevalence of co-morbidities and frailty, the severity of disability in this cohort is likely set to increase. As with frailty studies, there is much variation in the definition and measurement of disability in these studies with several different scores used, making comparison difficult and a consensus on definition and measurement is needed for future research in this area.

Strategies targeting potential modifiable risk factors for frailty, such as smoking cessation and physical activity, have yet to be shown to reduce disability in the setting of HIV but may be effective, such as has been demonstrated with smoking cessation and CVD events in PLWH [129]. However, given the heterogeneity in aetiology of disability in older PLWH, a multidisciplinary approach is likely to be required to reduce the burden of disability in this ageing cohort. 

### 4.6. Chronic Inflammation and Non-AIDS Illnesses

Chronic inflammation is thought to play a significant role in the development of non-AIDS illnesses in PLWH. Markers of systemic inflammation (hsCRP, interleukin 6 (IL6)) and coagulation (D-dimer) have been associated with all-cause mortality [130], non-AIDS illnesses (NAI) and more specifically cardiovascular disease (CVD) in PLWH [17,131,132,133]. Chronic inflammation is also associated with the development of CVD in the general population. The Veterans Aging Cohort Study Biomarker Cohort, which included both HIV^+^ and HIV^‒^ subjects, reported associations with IL6, D-dimer, soluble cluster of differentiation 14 (sCD14), a marker of monocyte/macrophage (M/M) activation, and mortality regardless of HIV status [134], while a Danish study also observed an association between higher levels of soluble CD163 (sCD163), another M/M activation marker, and all-cause mortality [135]. Although the majority of research to date has focused on the role of chronic inflammation in the pathogenesis of CVD in PLWH, as already discussed, markers of chronic inflammation have also been associated with the development of other NAI, including non-AIDS malignancies.

Control of HIV viremia with ART appears beneficial in reducing circulating markers of inflammation and coagulation [130,136,137], however disruption of M/M function, critical to the innate immune response, persists despite effective ART. A sub-study of a large, prospective, randomized trial of PLWH initiating ART demonstrated that levels of sCD163 and the proportion of ‘intermediate’ monocytes (CD14^+^ + CD16^+^) decreased with ART initiation while sCD14 did not decline and the proportion of pro-inflammatory, ‘non-classical’ monocytes (CD14 dim/- CD16^+^) actually increased [136]. 

A further study by our group measuring biomarkers reflecting endothelial (ICAM, V-CAM), platelet (GPVI, sCD40L) and M/M activation (sCD14, sCD163) pathways in a cohort of untreated HIV+ subjects initiating ART and HIV– controls matched for age, gender, ethnicity and smoking status, demonstrated that untreated subjects had elevated biomarkers reflecting activation in all three pathways, which partially normalized with ART. However, sCD14 remained persistently elevated despite effective ART [137] and these findings have since been replicated in a cohort initiating ART in early HIV infection [138]. ‘Non-classical’ monocytes have been associated with progression of coronary artery calcium on cardiac computed tomography angiography (CCTA) in PLWH [139] and innate immune activation, measured by elevations in sCD14 and sCD163, also associated with coronary artery disease [140] as well as progression of subclinical atherosclerotic plaques on CCTA [141]. 

**Figure 2:**
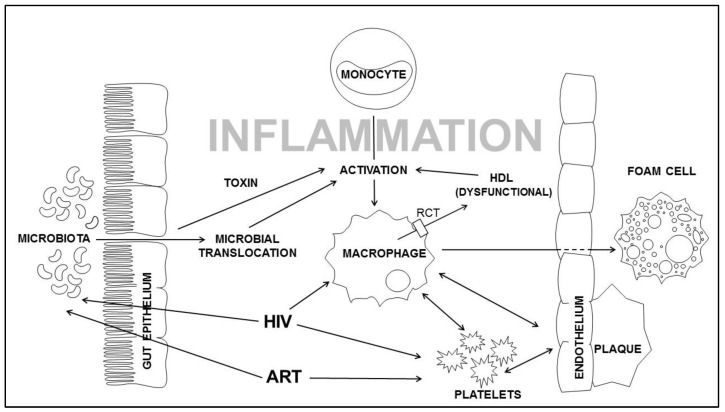
Inflammation and innate immune activation contribute to pathogenesis of CVD in PLWH.

The cause of this persistent innate immune activation in treated HIV infection is unclear but may be related to factors such as persistent low grade viral replication, viral co-infections (e.g. CMV) and persistent immune activation by gut microbial translocation. Loss of gut epithelial barrier integrity occurs early in HIV infection, with depletion of both T-cell and dendritic cells from the lamina propria of the gastrointestinal wall leading to translocation of gut microbial and metabolic products into the circulation which may contribute to a M/M inflammatory profile that has been shown to be associated with risk of atherosclerosis and CAD in the general population (Figure 2) [142,143]. 

In a prospective study of HIV+ subjects initiating ART, our group examined levels of intestinal fatty acid binding protein (I-FABP), a measure of gut barrier integrity and demonstrated an increase in I-FABP in the HIV+ group after ART initiation [137], a phenomenon replicated in a more recent study [138] suggesting despite effective ART and perhaps in part due to ART, microbial translocation and increased gut permeability persist.

The role of the composition of the gut microbiome to persistent inflammation in PLWH is still unclear, with microbiome studies in PLWH yielding inconsistent results and failing to identify a definitive dysbiosis that reflects HIV. However, studies have shown reduced bacterial microbiome diversity associated with more advanced immunosuppression, along with the expansion of adenoviruses and *Enterobacteriaecae,* both implicated in disruption of gut epithelial barrier function [144,145,146]. Recent studies have also associated microbiome dysbiosis with microbial translocation and immune activation [147,148] while studies from the general population have identified decreased microbial diversity linked to a number of age-related conditions [149,150] suggesting that the composition of the gut microbiota may also play a role in perpetuating the chronic inflammatory state and subsequent development of NAI.

Given the accumulating evidence of the role of chronic inflammation in the pathogenesis of NAI in PLWH and in particular, CVD, agents targeting inflammation are currently being investigated as possible therapeutic interventions. Placebo controlled trials to assess efficacy in preventing cardiovascular events in PLWH are ongoing using low dose methotrexate [151], a folic acid analogue and potent immunosuppressant widely used in the treatment of rheumatoid arthritis, pitavastatin [36], a HMG- CoA reductase inhibitor, which along with its lipid lowering properties, is also known to have anti-inflammatory properties by inhibiting intracellular isoprenoid formation and canakinumab [152], a novel anti IL-1beta monoclonal antibody, which is used in a number of rare inflammatory disorders and which has been shown to reduce recurrent cardiovascular events in the general population, although with an increased risk of serious infection [153]. What impact, if any, these interventions will have on the development of CVD in PLWH remains to be seen. Interestingly, an inverse association between use of HMG-CoA inhibitors or ‘Statins’ and non-AIDS cancers in PLWH has been reported [154,155], presumptively through its action as an anti-inflammatory. Larger studies are needed to further investigate this association and to further elucidate the role of inflammation on the pathogenesis of other NAI in PLWH. 

### 4.7. Implications and Limitations of Current Body of Evidence

With age related illness emerging as a major cause of morbidity and associated healthcare expenditure, in a globally ageing population of PLWH, pre-emptive planning is necessary in order to minimise the impact of comorbidities on patients’ health but also on healthcare services. Further research is necessary to develop effective preventative strategies, some of which have been discussed earlier, such as the use of anti-inflammatory agents, statin therapy and risk reduction interventions in PLWH with additional resources necessary in this area. Further research is also required to identify PLWH who may be at increased risk of NAI and who would maximally benefit from these interventional strategies, such as the identification of biomarkers or immunological factors that are associated with NAI. An inverted CD4^+^ CD8^+^ T cell ratio despite effective ART has been suggested as one such factor, however, the association with NAI has not been consistently seen and further large prospective studies are needed. 

It is necessary also to examine the current model of HIV care provision in the context of an ageing population. Traditional HIV clinics may not have the resources or the appropriate expertise to deal with these often-complex patients with multiple co morbidities. Some HIV care centres are providing dedicated clinics for elderly PLWH in which there is a multidisciplinary approach to their treatment, however, the optimal model of clinic service provision in this cohort has yet to be established and appropriate healthcare modelling and planning including expectant expenditure is necessary before role out of such clinics is widespread. 

While the potential implications of an ageing population of PLWH are evident, there are also, however, a number of limitations in the current research body regarding HIV and Ageing which limit their generality. The majority of studies in this area are conducted in European and North American populations with an over representation of males and PLWH of Caucasian ethnicity whereas, with the roll out of effective ART, the burden of age related comorbidities will likely be felt most in sub Saharan African countries and further studies in these populations are necessary. What impact, if any, current recommendations of commencing ART irrespective of CD4^+^ T cell count, with a presumptive beneficial effect on immunological function and reduction in both AIDS and non-AIDS related conditions in the short term, will have on the development of comorbidities in an ageing population needs to be clarified and further longitudinal studies are necessary to assess the long-term implications of these recommendations. 

## 5. Conclusions

With successful virological treatment of HIV, life expectancy for some persons living with HIV is approaching that of the general population. However, as an ageing cohort, PLWH are at increasing risk of comorbidities, frailty and disability at a potentially younger age than the uninfected population. Further research is needed to firstly define factors, both HIV and ART related, that specifically contribute to increased risk of comorbidities and frailty leading to disability, secondly to refine screening and diagnosis to take those HIV specific factors into consideration and lastly to identify interventions in order to reduce the excess burden of morbidity and mortality and allow successful ageing of people living with HIV.
